# The Frequency Response Characteristics of Ge-on-Si Photodetectors Under High Incident Power

**DOI:** 10.3390/nano15050398

**Published:** 2025-03-05

**Authors:** Jin Jiang, Hongmin Chen, Fenghe Yang, Chunlai Li, Jin He, Xiumei Wang, Jishi Cui

**Affiliations:** 1 Xiamen King Long United Automotive Industry Co., Ltd., Xiamen 361023, China; jj17@tsinghua.org.cn; 2 School of Information Engineering, Sanming University, Sanming 365004, China; chm@fjsmu.edu.cn; 3 Zhang Jiang Laboratory, Shanghai 201210, China; yangfh@zjlab.ac.cn; 4 Shenzhen Institute of Peking University, Shenzhen 518057, China; licl@ier.org.cn; 5 Shen Zhen SoC Key Laboratory, PKU-HKUST Shenzhen-Hongkong Institution, Shenzhen 518057, China; frankhe@pku.edu.cn

**Keywords:** photodetectors, high-power optical signals, bandwidth, carrier-shielding effect, carrier saturation drift velocity

## Abstract

This study explores the mechanisms responsible for the bandwidth reduction observed in germanium photodetectors under high signal light power. We investigate the impact of the carrier-shielding effect on the bandwidth through simulations, and we mitigate this effect by increasing the applied bias voltage. The increase in the concentration of photogenerated carriers leads to a reduction in the carrier saturation drift velocity, which reduces the bandwidth of the germanium photodetector; this phenomenon is studied for the first time. The bandwidth is determined primarily by the carrier saturation drift velocity when the incident light power is below 2.5 mW. The decrease in bandwidth that is calculated based on the decrease in carrier saturation drift velocity is consistent with the experimental results. However, when the signal light power exceeds 3 mW, both the carrier-shielding effect and the reduction in the carrier saturation drift velocity contribute to the bandwidth reduction. This study provides good theoretical guidance for the design of high-power germanium photodetectors.

## 1. Introduction

Silicon photonics has been applied to optical communication and interconnection, and its applications in optical computing, optical sensing, and lidar have attracted extensive research interest [1,2,3]. As germanium photodetectors are the key devices in the receiver, their performance plays a key role in the entire optical transceiver system. High responsivity and bandwidth have been needed for the development of photodetectors [4,5,6].

For applications such as coherent optical communication, lidar, and microwave photonics, photodetectors must possess both high optical signal power and high operating frequency [7,8]. Currently, there are several papers on germanium photodetectors that exhibit excellent frequency characteristics under high incident light power [9,10,11].

The bandwidth of germanium photodetectors can decrease under high incident light power, which limits their application [12,13,14]. The main reason for the decrease in bandwidth is the carrier-shielding effect described in previous papers [15,16,17]. To reveal the reasons for the bandwidth decrease under high incident light power, this paper conducted simulations and experiments on the carrier-shielding effect. To the best of our knowledge, this is the first time that the impact of the decrease in carrier saturation drift velocity, which is caused by a high concentration of photogenerated carriers, on the bandwidth of germanium photodetectors has been studied. This research provides theoretical support for the design of germanium photodetectors for high-power applications.

## 2. Structure and Process

**Figure 1:**
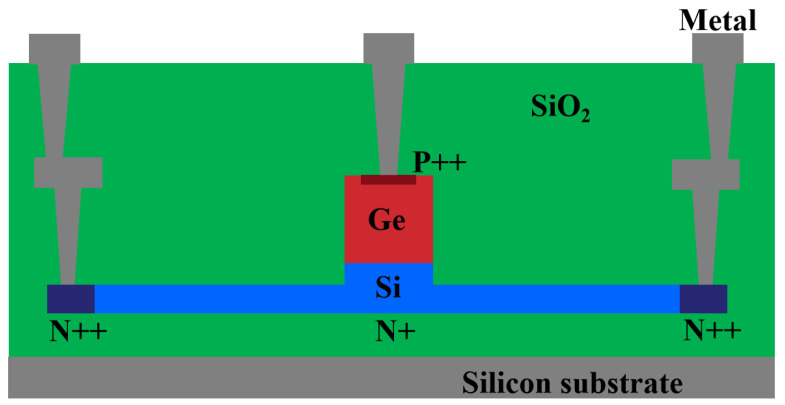
Cross-sectional diagram of the germanium PIN photodetector.

Figure 1 presents a cross-sectional diagram of a vertical PIN germanium photodetector. This device is fabricated on a silicon on insulator (SOI) substrate, where the silicon layer functions as both the slab and the waveguide regions. The silicon slab consists of both lightly and heavily doped areas. The lightly doped region, spanning from the rib waveguide to the electrode, is 1.5 μm wide and has a doping concentration of 1 × 10^−17^ cm^−3^. To ensure optimal ohmic contact between the slab and the metal electrode, a heavily doped contact region with a doping concentration of 1 × 10^−20^ cm^−3^ is incorporated. The thickness of the silicon slab is 60 nm. The width of the 220 nm thick rib waveguide is 1.5 μm and the length is 10 μm. The doping concentration for the silicon rib is 1 × 10^−15^ cm^−3^; this concentration ensures the transport of high-concentration photogenerated carriers without significant light absorption. The germanium absorption layer is epitaxially grown on the silicon rib waveguide region with a thickness of 600 nm, and its width and length match those of the silicon rib waveguide region. For ohmic contact with the electrode, the top of the germanium absorption layer is heavily doped with a concentration of 3.8 × 10^−18^ cm^−3^.

The fabrication process begins with an SOI wafer, which then has its top layer of silicon etched, on which the non etched area is the silicon waveguide and the shallow etched area is the slab region. Next, the wafer undergoes a two-step boron implantation process that involves a light implantation followed by a heavy implantation to form p-type ohmic contacts. The dopants within the silicon are then activated using a rapid thermal annealing (RTA) process at 1030 °C for 5 s, which precedes the germanium epitaxy step. Subsequently, phosphorus ion implantation is carried out on the germanium layer to create the n-type ohmic contact region. Following the deposition of the cladding oxide, the fabrication process concludes with the formation of contact vias and the patterning of aluminum interconnects. The defect density of the top silicon layer in the SOI is 3 × 10^4^ cm^−2^ and the defect density of the epitaxial germanium layer is 7 × 10^4^ cm^−2^.

An increase in the size of the germanium absorption region improves the level of high incident light power processing of the photodetector. However, it increases the device’s capacitance, which reduces the bandwidth of the device.

The 3 dB bandwidth of the photodetector was measured using a setup comprising a vector network analyzer (VNA, KEYSIGHT PNA-X Network Analyzer N5247A, Keysight, Santa Rosa, CA, USA) and a high-performance LiNbO_3_ modulator with a 40 GHz bandwidth. A precision DC power supply (KEITHLEY 2611A SYSTEM Source Meter, Keithley, Santa Rosa, CA, USA) was used to apply the DC bias voltage to the photodetector through a bias tee.

## 3. Experiments and Discussion

**Figure 2:**
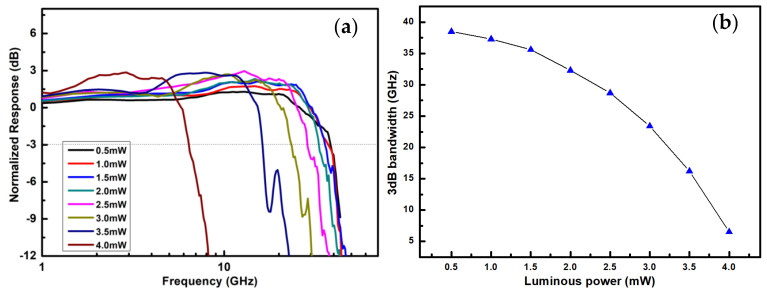
(**a**) Normalized S_21_ traces of the photodetector under different incident light power levels. (**b**) The 3 dB bandwidths of the photodetector under different incident light power levels.

We experimentally tested the bandwidth characteristic curves of the photodetector under various incident light powers at a −1 V bias voltage, as shown in Figure 2. The noise generated by the device and the circuit’s jitter can lead to testing inaccuracies. To mitigate this, we performed a full calibration of the entire system prior to conducting the tests. The vector network analyzer emits electrical signals, which are amplified and loaded onto a 40 GHz bandwidth lithium niobate modulator. The modulated optical signal enters the detector and is converted into an electrical signal, which is transmitted back to the vector network analyzer. Before testing, it is necessary to calibrate the entire link system to deduct errors generated by the instrument. A 1550 nm laser was utilized; all the lasers mentioned in this paper operate within this wavelength range. To facilitate a clear understanding of the relationship between the bandwidth and the incident light power, power is expressed in milliwatt (mW) units. The minimum incident light power was set to 0.5 mW, with increments of 0.5 mW, resulting in the following power levels: 0.5 mW, 1 mW, 1.5 mW, 2 mW, 2.5 mW, 3 mW, 3.5 mW, and 4 mW. The corresponding optoelectronic bandwidths for each incident light power were determined to be 38.56 GHz, 37.33 GHz, 35.62 GHz, 32.34 GHz, 28.75 GHz, 23.41 GHz, 16.26 GHz, and 6.52 GHz, respectively, as shown in Figure 2. The experimental results demonstrate that the bandwidth decreases with increasing incident light power and that the rate of the decrease becomes more pronounced at higher power levels. The relationship between the bandwidth and the incident light power is further illustrated in Figure 2b. As optical power increases, the responsivity follows a two-stage behavior. In the first stage, the responsivity remains linear, with a value of 1.13 A/W when the incident optical power is ≤4.37 mW. However, when the incident optical power exceeds 4.37 mW, the responsivity gradually decreases with increasing optical power. Also, the rate of decrease becomes progressively slower. The absorption conversion efficiency of this photodetector is 90.4%. The main causes of light loss include the reflection and scattering of light at the silicon/germanium heterojunction, the absorption of signal light by the metal electrodes, and defects and impurities in the germanium material, which promote the recombination of photogenerated carriers. At a bias voltage of −1 V, the dark current of the detector is 10.32 nA. We used a commercially available modulator to modulate the transmitted pseudo-random sequence code elements and measured a bit error rate of 1.23 × 10^−6^ for the photodetector.

A major factor contributing to the reduction in bandwidth at high incident light power is the carrier-shielding effect [12,13,14]. This carrier-shielding effect occurs due to the rearrangement of photogenerated carriers under the influence of an external electric field. Photogenerated holes move in the direction of the applied electric field, whereas photogenerated electrons move in a direction opposite to the applied electric field. Then, the electrons and holes generate an internal electric field in the opposite direction to that of the external electric field. When the built-in electric field is superimposed with the external electric field, the electric field strength in the absorption region is weakened by the built-in electric field. When the concentration of photogenerated carriers is high, this effect is more pronounced. The carrier-shielding effect leads to a decrease in the electric field strength in the absorption region, thereby reducing the migration rate of the carriers.

**Figure 3:**
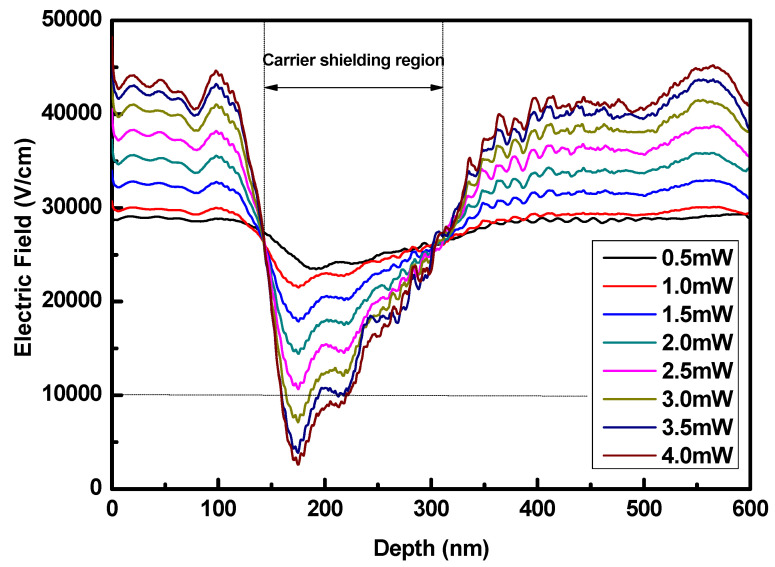
The magnitude of the electric field in the germanium layer is measured along the metal contact and the silicon waveguide at a point 1.1 µm from the front edge of the absorber and at the center along the width.

To quantitatively study the effect of carrier shielding on the bandwidth, we simulated the distribution of the internal electric field at different incident light power levels with the finite-difference time-domain (FDTD) method. The results are shown in Figure 3. The simulation boundary conditions are set to the perfectly matched layer (PML), with a mesh size of 1 nm. The convergence of the FDTD method relies on the careful selection of step size, appropriate boundary condition settings, and effective error control. The carrier-shielding effect occurs in the light field distribution region within the germanium absorption area, and its intensity is directly proportional to the concentration of photogenerated carriers. Its maximum value is located about 1 μm from the front end of the germanium absorption region [18]. Therefore, the simulation is focused on the 1D electric field intensity in the germanium absorption region between the metal electrode and the silicon waveguide at this position. When a bias voltage of −1 V is applied, the minimum internal electric field intensity is approximately 2.34 × 10^4^ V/cm at an incident light power of 0.5 mW. As the incident light power increases to 4 mW, the minimum internal electric field intensity decreases significantly to 0.26 × 10^4^ V/cm. This indicates that the electric field in the photogenerated carrier region decreases substantially, whereas the field outside the carrier region increases. These results suggest a pronounced carrier-shielding effect in the region where the electric field weakens.

The electric field intensity required to reach the saturation drift velocity of charge carriers in germanium is 0.97 × 10^4^ V/cm [19]. When the incident light power is less than 2.5 mW, although the carrier-shielding effect reduces the local electric field intensity, it still allows the carriers to reach their saturation drift velocity. In other words, when the incident light power is below 2.5 mW, the decrease in electric field intensity due to the carrier-shielding effect does not impact the device bandwidth. However, when the incident light power exceeds 3.0 mW, the carrier-shielding effect begins to reduce the bandwidth.

**Figure 4:**
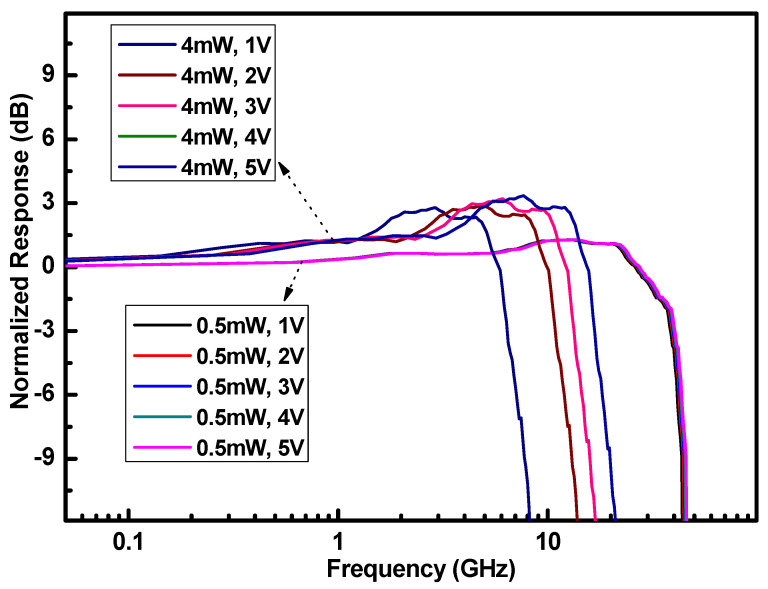
Normalized S_21_ response of the photodetectors under different bias voltages at incident light powers of 0.5 mW and 4 mW.

The carrier-shielding effect can reduce the electric field intensity in the photodetector at high incident light power levels. To counter this, we can increase the device’s internal electric field intensity by increasing the bias voltage, thereby reducing or even eliminating the impact of carrier-shielding on the device bandwidth. The experimental results are presented in Figure 4.

We applied voltages of −2 V, −3 V, −4 V, and −5 V for incident light powers of 0.5 mW and 4 mW, and we compared the resulting bandwidth characteristics to those observed at a −1 V bias. Given that the photodetector’s reverse breakdown voltage is −6 V, those test voltages were set within an appropriate range. At an incident light power of 0.5 mW, increasing the applied bias voltage from −1 V to −5 V resulted in almost no change in bandwidth. However, at an incident light power of 4 mW, increasing the bias voltage to −2 V, −3 V, −4 V, or −5 V increased the bandwidth to 10.84 GHz, 15.03 GHz, 18.62 GHz, or 18.62 GHz, respectively. When the bias voltage is increased to −5 V, the bandwidth characteristics remain almost consistent with those at −4 V and do not continue to increase. The test was conducted by only changing the voltage while keeping the link unchanged so that the bandwidth curves at −5 V and −4 V overlap. This observation is consistent with the simulation results shown in Figure 3. When the incident light power is 4 mW, the simulation results in Figure 3 show that the light absorption region has a significant carrier-shielding effect. We eliminated the carrier-shielding effect by increasing the bias voltage during the experiment, and the bandwidth of the photodetector was significantly improved. When the incident light power is 0.5 mW, the simulation results of the absorption region electric field show that the carrier-shielding effect does not influence the carrier saturation drift velocity. In the experiment, by increasing the bias voltage to eliminate the carrier-shielding effect, the bandwidth of the photodetector remains almost unchanged. The simulation results and experimental results show good consistency.

When the bias voltage is −5 V and the optical power is 4 mW, the photodetector bandwidth reaches 18.62 GHz, which remains lower than the 38.56 GHz bandwidth observed at an optical power of 0.5 mW with a −1 V bias. These experimental results indicate that at high incident light power, the carrier-shielding effect reduces the device bandwidth by lowering the electric field intensity. However, the impact on the bandwidth is limited and is significant only when the incident light power is less than 2.5 mW. Both high optical power and bias voltage can compromise the device’s reliability.

The increase in carrier concentration at high incident light power leads to a decrease in the carrier saturation drift velocity [20,21], which is another key factor that contributes to the reduction in bandwidth. A higher carrier concentration increases the scattering probability during carrier drift, thereby decreasing the carrier saturation drift velocity. An increase in the carrier concentration leads to an increase in the scattering probability, which mainly includes scattering between electrons and holes, carriers and lattices, phonons, and defects [22,23]. The scattering between electrons and holes primarily arises from their interaction through Coulomb force, leading to a change in the carriers’ direction of motion. Phonon scattering occurs due to collisions between lattice vibrations and moving carriers, resulting in dispersion. Additionally, defects and impurity centers may capture carriers in motion, thereby diminishing their velocity. By lowering carrier concentration, operating at reduced temperatures, and minimizing impurities and defects, one can effectively decrease carrier scattering and enhance the migration rate of carriers nearing saturation. Intervalley scattering reduces the carrier saturation migration rate, thereby decreasing the device’s bandwidth. Phonon-mediated relaxation leads to an increase in scattering between phonons and carriers, which in turn lowers the mobility and reduces the device’s bandwidth. The scattering during carrier migration changes the direction of carrier movement, reducing the rate of carrier migration. Therefore, the rate of carrier movement along the electric field direction is reduced.

**Figure 5:**
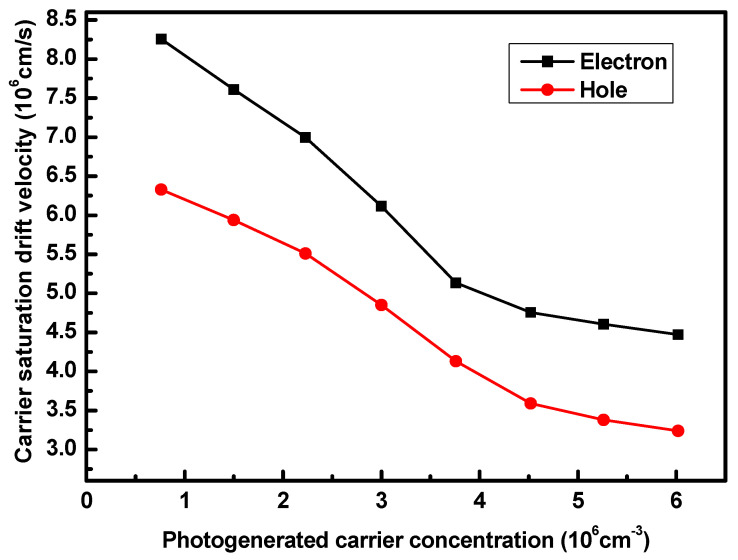
Carrier saturation drift velocity versus photogenerated carrier concentration in the germanium layer according to the measured Hall effect.

We experimentally measured the changes in the carrier saturation drift velocity at various photogenerated carrier concentrations using the Hall effect, as shown in Figure 5. The carrier saturation drift velocity in germanium was measured with an Ecopia HMS-3000 Hall tester (Ecopia, Anyang-city, Republic of Korea). The sample was clamped onto a sample plate with a probe and injected with light through an optical fiber. At incident light powers of 0.5 mW, 1 mW, 1.5 mW, 2 mW, 2.5 mW, 3 mW, 3.5 mW, and 4 mW, the corresponding concentration of photogenerated carriers in the high-field area of the germanium absorption region are 0.38 × 10^16^ cm^−3^, 0.75 × 10^16^ cm^−3^, 1.12 × 10^16^ cm^−3^, 1.51 × 10^16^ cm^−3^, 1.88 × 10^16^ cm^−3^, 2.26 × 10^16^ cm^−3^, 2.63 × 10^16^ cm^−3^, and 3.01 × 10^16^ cm^−3^, respectively. Since the electron saturation drift velocity is higher than that of the holes, we focus primarily on the effect of a reduced hole saturation drift velocity on the device bandwidth. The corresponding hole saturation drift velocities are 6.33 × 10^6^ cm/s, 5.94 × 10^6^ cm/s, 5.51 × 10^6^ cm/s, 4.85 × 10^6^ cm/s, 4.13 × 10^6^ cm/s, 3.59 × 10^6^ cm/s, 3.38 × 10^6^ cm/s, and 3.24 × 10^6^ cm/s.

The 3 dB bandwidth of the photodetector can be calculated from the RC constant and the carrier transport time [24,25]. The frequency formula corresponding to the carrier transport time is the following:

(1) $$f_{tr}=\frac{0.45v_{h}}{d},$$

where *v_h_* is the saturation hole drift velocity of the germanium and *d* is the thickness of the intrinsic germanium film. The frequency formula corresponding to the *RC* is the following:

(2) $$f_{RC}=\frac{1}{2\pi RC},$$

where *R* is the load resistance and *C* is the capacitance. The 3 dB bandwidth of the photodetector can be calculated as follows:

(3) $$f_{3dB}=\frac{1}{\sqrt{{f_{t_{r}}}^{-2}+{f_{RC}}^{-2}}}$$

**Figure 6:**
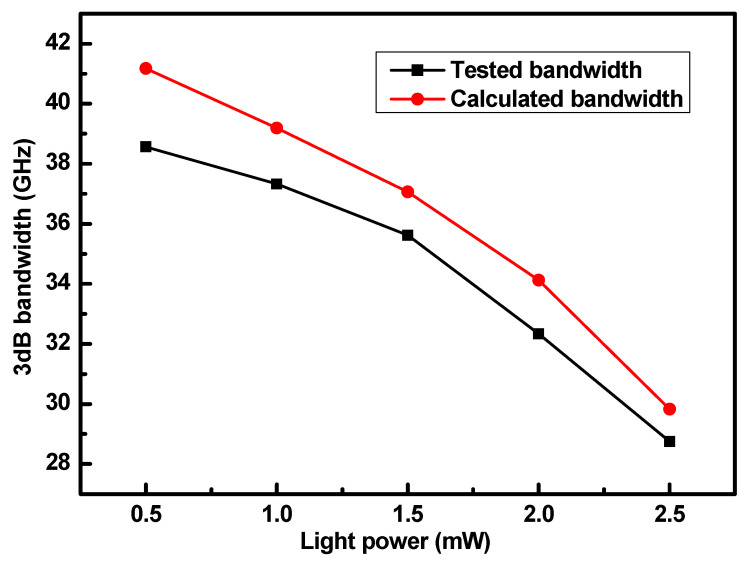
The tested and calculated normalized S_21_ response of the photodetectors under different incident light powers.

The measured series resistance was 618 Ω and the capacitance was 4.5 fF, resulting in an RC-limited bandwidth of 57.23 GHz. Using the saturation hole drift velocity obtained from the Hall effect measurements experimentally, we calculated the bandwidth at various optical powers, as shown in Figure 6. This experimentally determined bandwidth serves as a reference and is consistent with the data presented in Figure 2. The bandwidth derived from the saturation hole drift velocity aligns closely with our experimental results confirming that, for incident light powers less than 2.5 mW, the saturation hole drift velocity is the primary factor that influences the bandwidth. Notably, bandwidth values that are calculated using theoretical formulas tend to be slightly higher than those that are observed experimentally. This discrepancy is primarily due to losses and measurement errors during the testing process, which may affect the accuracy of the results. Overall, our findings highlight the critical role of the saturation hole drift velocity in determining photodetector performance under specific optical power conditions.

Under high incident optical power, the operating temperature of the photodetector will increase. The increased temperature will enhance lattice vibrations, thereby increasing the probability of carrier scattering, leading to a decrease in carrier saturation drift velocity.

## 4. Conclusions

This work investigated the mechanism underlying the bandwidth reduction in germanium photodetectors at high incident light power. According to experimental and simulation results, we conclude that the primary reasons for bandwidth reduction at high incident light power are the carrier-shielding effect and the decrease in the carrier saturation drift velocity. The carrier migration rate increases with the increase in electric field strength until it reaches the saturation mobility. The carrier-shielding effect reduces the electric field inside the absorption region, leading to a decrease in the carrier migration rate. The reduction in the saturation carrier mobility, which is the maximum migration rate of carriers, determines the highest rate at which carriers can migrate. We conducted multiple tests on the data in the paper and the trend of the test results shows good consistency. Each data point used in our paper is the median of multiple tests.

For incident light powers below 2.5 mW, despite a notable carrier-shielding effect, the electric field intensity across the absorption region remains sufficient for carriers to reach their saturation drift velocity. Thus, the carrier-shielding effect has a minimal impact on the bandwidth of the photodetector in this range. Instead, the primary factor influencing bandwidth is the increased concentration of photogenerated carriers, which leads to a reduction in the saturation drift velocity of the holes.

When the incident light power exceeds 3 mW, the electric field intensity in the carrier-shielding region becomes too weak to maintain the saturation drift velocity of the carriers. By increasing the applied bias voltage, we mitigate the effect of carrier shielding on the electric field intensity. However, the bandwidth remains lower than that observed at 0.5 mW. Therefore, an increase in the concentration of photogenerated carriers leads to a decrease in the saturation drift velocity of the holes, which is another significant factor in bandwidth reduction.

The main reasons for the decrease in bandwidth under high incident light power are the carrier-shielding effect and the decrease in carrier saturation drift velocity, both of which are caused by excessively high distributions of local carrier concentration. Therefore, in the design of germanium photodetectors, owing to the coupling of evanescent waves, the light field in the germanium absorption region exhibits a strong–weak alternating distribution. This leads to a carrier-shielding effect and a decrease in the carrier saturation drift velocity in regions with strong light field distributions, thereby affecting the bandwidth. Optimizing the structural design to make the light field distribution more uniform can effectively improve the frequency response of germanium photodetectors under high incident light power. The photodetector presented in this study did not demonstrate excellent saturation performance. The saturation characteristics of the device could be further improved by optimizing its design to enhance the uniformity of the light field distribution within the germanium absorption region.

## Data Availability

The original contributions presented in this study are included in the article. Further inquiries can be directed to the corresponding author.
